# Motivation Profile of Youth Greco-Roman Wrestlers; Differences According to Performance Quality

**DOI:** 10.3390/sports11020043

**Published:** 2023-02-09

**Authors:** Kreso Skugor, Barbara Gilic, Marijana Mladenovic, Valdemar Stajer, Roberto Roklicer, Kristijan Slacanac, Domagoj Bagaric, Hrvoje Karnincic

**Affiliations:** 1 Faculty of Kinesiology, University of Split, 21000 Split, Croatia; 2 College of Sports and Health, University of Belgrade, 11000 Belgrade, Serbia; 3 Faculty of Sport and Physical Education, University of Novi Sad, 21000 Novi Sad, Serbia; 4 Ministry of Tourism and Sport, 10000 Zagreb, Croatia; 5 Faculty of Kinesiology, University of Zagreb, 10000 Zagreb, Croatia

**Keywords:** athletes, combat, competition, self-determination theory, sport psychology

## Abstract

Athletes have to possess high motivation levels to perform each training session and competition at the highest level. Thus, the motivation of the wrestler is essential to reach the highest performance quality. The research included 47 Greco-Roman wrestlers aged 17.71 ± 1.62 years. Variables included anthropometric indices, sports motivation assessed by the revised Sport Motivation Scale (SMS-II), and competitive success (medal winners and non-winners at the National Championship). The Cronbach’s alpha coefficients checked the internal consistency of the SMS-II. Differences between performance quality were determined by Cohen’s d effect sizes, and MANOVA for motivation and anthropometric variables/body build variables. In the total sample, wrestlers had high levels of intrinsic motivation (5.97 ± 0.90), integrated (5.99 ± 0.83), and identified (6.08 ± 0.82) regulation, while they had low amotivation (2.53 ± 0.98) and external regulation (3.26 ± 1.24). Successful wrestlers had significantly higher intrinsic motivation than less successful wrestlers (Cohen’s d = 0.76, moderate effect size). Results evidenced that wrestlers have high self-determined motivation, which is vital for maximal performance and persisting in sports. Future research should investigate wrestlers from other age groups to ultimately determine the sport motivation profile of wrestlers and enable their optimal sports development.

## 1. Introduction

Successful wrestlers have to possess high levels of physical and psychological readiness as wrestling is deemed to be an extremely demanding sport [1,2]. Indeed, a wrestling match consists of two rounds lasting three minutes with only a 30 s break between the rounds. During the match, wrestlers are constantly performing high-intensity attack and defense maneuvers in the submaximal and maximal physiological zones [3]. Thus, wrestlers must possess highly developed anaerobic capacities, and the ability to endure high energy demands during the entire match [4]. Numerous studies that investigated success factors of wrestlers identified that anaerobic power, strength endurance, upper and lower body strength, and technical skills, such as a throw over the hip and a supplex throw differentiate successful from less successful wrestlers [5].

When observing complete athletic performance and determinants that are important for identifying athletic talents, studies are contradictory as some consider that sports success is genetically predetermined, while others believe that highly motivated practice leads to sports success. A review study that aimed to collect studies regarding precursors and prerequisites of athletic talent and sports success noted that apart from appropriate body status and sport-specific abilities (i.e., strength, endurance, and explosiveness), psychological factors perform an essential role in becoming a top-performance athlete [6]. Specifically, future champions are characterized by their attitude to training and performing more frequently longer and harder training routines (hard work ethics) [6,7,8]. Moreover, studies noted that dedication, determination, persistence, and intrinsic motivation have the predictive potential of athletic talents [6]. When observing combat athletes, they reached the highest sports results (world-class status) after accumulating 3000–7000 h of specialized preparation through 4–7 years [6]. This could all lead to the assumption that psychological characteristics perform a crucial role in creating successful athletes.

Researchers mainly strive to find out how to affect and predict performance. Hence, sports psychologists aim to determine performance predictors, with mood states being one of the leading research topics. Specifically, the relationship between the profile of mood states (POMS) and sports performance has been extensively examined over more than 50 years since it was shown that it could predict sports outcomes [9]. In that manner, it was noted that successful athletes had lower tension, depression, anger, confusion, and fatigue, while they had higher vigor than less successful athletes [10]. Moreover, it is proposed that POMS subscales have greater predictive power for sports success in short-duration and individual sports [9]. For example, it was shown that anger could improve performance in short-duration sports, such as judo, karate, and wrestling [11]. All the aforementioned regarding POMS research could be related to explaining the psychological background that determines success in sports performance, which is described in more detail in further text.

Regarding the fact that wrestling is an extremely physically demanding sport, it is logical to assume that to endure high physical requirements, wrestlers must also have a highly developed psychological component. Additionally, psychological factors are essential as wrestling is a sport where two athletes perform in order to achieve a superior position using both body and intelligence [12]. What is essential is that wrestlers must possess high levels of motivation to perform each training session and competition at the highest level [13]. Additionally, competitors rely on themselves; therefore, their motivation to perform is responsible for reaching competitive success. Motivation is defined as “the direction and intensity of action“ and includes factors influencing behavior [14]. Motivation enables an individual to be attracted to the targeted activity and depicts why people act the way they do in specific situations [14].

In a sports context, motivation relates to the presence of processes and factors which stimulate athletes to be active or inactive in different situations. The self-determination theory (SDT) is the most complete and stable framework regarding sport motivation in various contexts, as it emphasizes the necessity of the drive influenced by the need for autonomy and self-actualization [15]. SDT states that athletes are usually motivated by either external factors (e.g., rewards, pressure from opponents, and peoples’ opinions) or internal factors (e.g., curiosity, desire to grow, and self-improvement) [16]. Additionally, it describes how different motives are associated with involvement in physical activity and sports in different ways [17]. SDT depicts motivation as a continuum ranging from amotivation (lack of motivation) through extrinsic (controlled) motivation to intrinsic motivation, which is the most self-determined and autonomous.

According to the SDT, a Sport Motivation Scale (SMS) has been constructed [18], and recently, a revised scale named SMS-II has been validated and gained increased research use in various sports [15,19]. SMS-II consists of six subscales, ranging from amotivation (least self-determined type of motivation), external motivation, which includes external, introjected, identified, and integrated regulation, to the most self-determined type of motivation: intrinsic motivation [15]. The least self-determined type of behavior regulation is the amotivation, which relates to the state where an individual does not have any impetus to act. Furthermore, extrinsic motivation consists of four constructs: (i) external regulation; (ii) introjected regulation representing the controlling and least self-determined construct of extrinsic motivation because they represent motivation to obtain external rewards (e.g., money, medals) and avoid punishments; (iii) identified regulation which is not entirely external, where behavior is commenced out of choice; and (iv) integrated regulation, the most self-determined type of extrinsic motivation, that describes personally endorsed behaviors becoming coherent, assimilated, and integrated within the self. Finally, the most self-determined and autonomous form of motivation is intrinsic motivation, which is based on the drives of satisfaction and joy [15]. Intrinsic motivation leads to voluntary participation in the sports activity with an absence of external pressures and rewards, which means that participation in the activity is for the satisfaction, fun, interest, and pleasure [18]. Therefore, it was hypothesized that athletes with higher intrinsic motivation would be more persistent and committed to sport which leads to better sports performance.

SDT has been confirmed as an appropriate framework for understanding and promoting motivation in sports [20,21]. Therefore, SDT could also be interesting for observing the sport motivation profile of wrestlers. Indeed, it could be assumed that motivation is highly important for success in wrestling competitions. Therefore, the present study aimed to determine (i) the sport motivation profile of youth wrestlers and (ii) differences in motivation according to the quality of wrestlers (i.e., competitive success). The results of this study could help coaches and practitioners elucidate the motivation profile of their athletes, which could help develop the wrestler’s optimal performance.

## 2. Materials and Methods

### 2.1. Participants

This study included 47 Croatian Greco-Roman wrestlers aged 17.71 ± 1.62 years with a training experience of 6.89 ± 2.75 years. The calculated required sample size was 37 wrestlers, as the overall population of young wrestlers that participated in National Championship was 170 (cadets and juniors). The inclusion criteria were at least two years of structured wrestling training and participating in National competitions. This way, the authors wanted to make sure that included wrestlers have experience and developed a psychological profile that relates to this particular sport. The exclusion criteria were any illness or injury over the last two months, or any other condition and situation that could have prevented wrestlers from regular training and providing their maximum during the tests. Participants were informed about the study procedures before the initiation of the study, and participants or their legal guardians (for participants under the age of 18) signed informed consent. The study was approved by the Ethical Board Faculty of Kinesiology, University of Split (Ref.no. 2181-205-02-05-22-0012).

### 2.2. Variables and Procedures

Variables included demographic characteristics (age and gender), anthropometric indices, sports motivation, and competitive success.

Anthropometric variables included body mass, body height, and percentage of body fat. The percentage of body fat was determined by the Slaughter-Lohman formula calculated from a sum of triceps and calf skinfolds measured by Harpenden skinfold caliper (British Indicators, Burgess Hill).

Sports motivation was assessed by the revised sport motivation scale (SMS-II) which was translated into the Croatian language. SMS-II consists of 18 items, forming six subscales depicting low to a high level of autonomy (amotivation, external, introjected, identified and integrated regulation, and intrinsic motivation). The SMS-II was previously indicated as valid and reliable for assessing sports motivation [15]. The reliability and validity of the Croatian version of the SMS-II scale was assessed on athletes from different sports (unpublished material). The motivation was evaluated on the 7-point Likert scale, from 1, meaning “Does not correspond at all,” to 7, “Corresponds completely.”

Competitive success was determined based on the results (competition rankings) from the last National championship held in 2022. According to the competition rank, wrestlers were divided into two categories: (i) successful wrestlers (medal winners at the National Championship), and (ii) less successful wrestlers who did not win a medal at the National Championship.

### 2.3. Testing Protocol

All assessments were conducted prior to the training sessions when athletes were rested and relaxed. First, anthropometric indices have been measured. Then, investigators explained the aim of the questionnaire they were about to fulfill. Participants fulfilled the SMS-II on their mobile phones (or investigators gave the devices to athletes that did not have it). The SMS-II was fulfilled on the online platform SurveyMonkey, which enabled investigators to collect responses directly in a digital shape (i.e., excel sheet).

### 2.4. Statistical Analysis

The normality of the variables was checked by the Kolmogorov–Smirnov test. Descriptive statistics included means and standard deviations for all variables.

The internal consistency (reliability) of the six SMS-II subscales was checked by Cronbach’s alpha coefficients. Cronbach’s alpha coefficients indicate the correlation between items, meaning that appropriately high values justify categorizing items into a subscale. Values lower than 0.5 were considered unacceptable, 0.5–0.60 as poor, 0.60–0.70 as questionable, 0.70–0.80 as acceptable, 0.80–0.90 as good, and >0.90 as excellent [22]. Additionally, the Inter-item correlation coefficients were calculated to support the internal consistency of the SMS-II additionally.

The discriminative validity of the SMS-II was determined by comparing two performance levels (i.e., quality groups of wrestlers). The differences between performance levels were checked in several ways. First, differences between performance levels (successful vs. less successful wrestlers) were assessed by the magnitude-based Cohen’s effect sizes with modified qualitative descriptors. The effect size was evaluated based on the following criteria: <0.02 represented trivial; 0.2–0.6 represented small; >0.6–1.2 represented moderate; >1.2–2.0 represented large; and >2.0 represented very large differences. Moreover, differences in the motivation variables and anthropometric/body composition variables were calculated using the multivariate analysis of variance (MANOVA). Package Statistica 13.5 (Tibco Inc., Palo Alto, CA, USA) was used for all statistical calculations, with an applied *p*-level of 0.05.

## 3. Results

SMS-II displayed appropriate internal consistency (Table 1). Cronbach’s alpha coefficients ranged from unacceptable to good (0.33–0.79). Even though some subscales (Introjected regulation and amotivation) had unacceptable and poor Cronbach’s alpha values, they were retained for the analysis because these subscales had adequate reliability in previous research, in which it was also proved that the Cronbach’s alpha coefficients would not increase if any of the items were deleted [15].

Descriptive statistics and differences between successful and less successful wrestlers are shown in Table 2. Successful and less successful wrestlers did not differ in age, training, competing experience, or anthropometric variables. A very large effect size was noted for competition ranking, meaning that successful and less successful wrestlers had significant differences, and were not close in the ranking (i.e., there was a possibility that the difference is low, and that the non-medal winners were for example on the fourth place).

**Figure 1:**
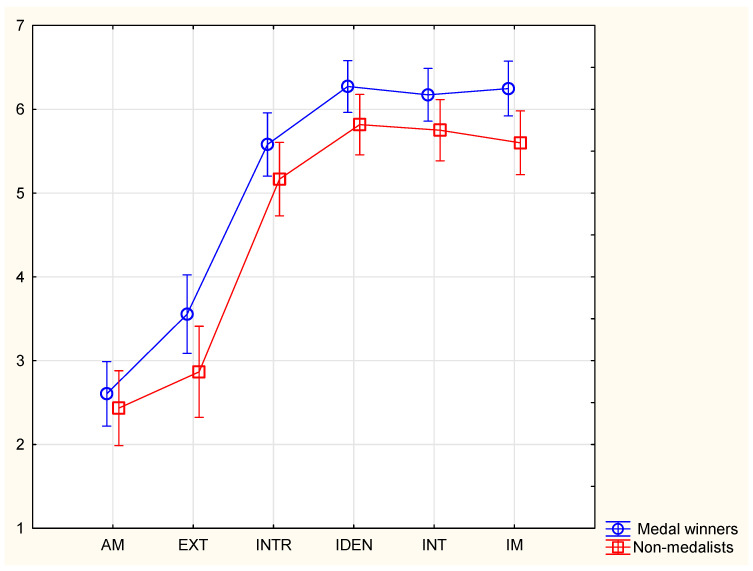
Motivation profile of successful and less successful wrestlers. (Note: AM—amotivation, EXT—external regulation, INTR—introjected regulation, IDEN—identified regulation, INT—integrated regulation, IM—intrinsic motivation).

The graphical presentation of the sport motivation profile is displayed in Figure 1. It is visible that, on a motivation continuum, wrestlers have high levels of self-determined forms of motivation, and low levels of amotivation and external regulation. Additionally, successful wrestlers have significantly higher levels of intrinsic motivation compared to less successful wrestlers (6.25 ± 0.75 vs. 5.60 ± 0.96, moderate effect size).

Multivariate analysis of variance (MANOVA) did not reveal differences between medal winners and non-winners in motivation variables nor in anthropometric/body composition variables (Table 3).

**Figure 2:**
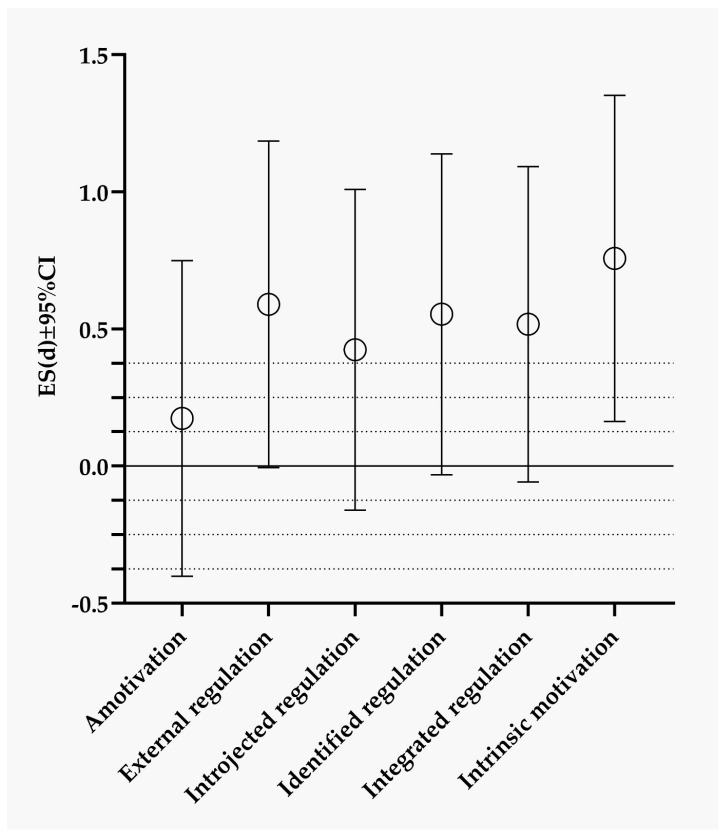
Differences between successful and less successful wrestlers in the motivation variables displayed through effect sizes.

When further observing motivation variables on the subscales, there were differences in the intrinsic motivation between successful and less successful wrestlers (moderate effect size) (Figure 2).

## 4. Discussion

This study aimed to determine the motivation profile of youth wrestlers and investigate the differences between performance categories. The most important findings of this research are: (i) wrestlers have high levels of self-determined motivation and low amotivation, and (ii) successful wrestlers have higher intrinsic motivation compared to less successful wrestlers.

### 4.1. Motivation Profile of Greco-Roman Wrestlers

Results evidenced that wrestlers possess high levels of self-determined forms of motivation (i.e., intrinsic motivation and integrated regulation), while external regulation and amotivation were low. In sports, especially in competitive sports, the more self-determined and autonomous types of motivation have been linked to long-time commitment and greater interest in sports participation at a high level [15]. Indeed, a predominance of autonomous and self-determined types of motivation in our wrestlers can be explained similarly. Wrestlers exclusively rely on themselves, and their goal is to perform constantly on the maximal level. Additionally, wrestlers have to put a lot of time and effort into their training sessions to become superior to their opponents and achieve their top performance. Thus, they have to possess a quality (i.e., motivation) that will allow them to execute extremely demanding training and competitions. Indeed, motivation has a direct impact on sports success as it regulates effort and time, which is necessary to overcome competitive challenges and obtain goals [23]. It is important to emphasize that more self-determined motivation leads to more positive consequences. Precisely, athletes with more self-determined motivation would have better objective performance, cope with challenges, put in more effort, decrease burnout, and have better mental health [24,25].

Additionally, individuals with more autonomous motivation will more likely continue practicing their sport and will have decreased chances of dropping out from sport. Indeed, a study on athlete students noted a positive relationship between the intention to continue participating in sports and autonomous motivation [26]. Similarly, a study on young athletes from different sports found that intrinsic motivation leads to adherence to sports practice [27]. Having that in mind, a recent study that investigated volleyball players noted a positive relationship between autonomous motivation and enjoyment, which most likely keeps athletes to continue practicing their sport [28]. Indeed, a study on Hungarian adolescent athletes that investigated commitment and motivation recorded a positive association between intrinsic motivation and sport commitment (i.e., enthusiastic commitment) [29]. Therefore, autonomous motivation not only leads to athletes being more committed and strive for better results due their personal satisfaction, but it also influences persistence in sports. We can theorize that persistence could be connected with autonomous motivation as athletes are actually enjoying, having fun and are satisfied with themselves while practicing sports.

Several studies supported our findings that wrestlers possess self-determined forms of motivation. A study on Bulgarian national team wrestlers aged 16–35 years recorded that wrestlers have higher intrinsic than extrinsic motivation [30]. Supportively, Spanish Olympic wrestlers reported that motivation is the construct that guides them to success. Specifically, wrestlers consider that their intrinsic motivation ensures overcoming several daily training sessions (i.e., 2–3 training sessions a day), and is the catalyst for the sport’s painful and challenging character [31]. Wrestlers reported that the most important reason for training and competing is to become the winner [31]. Indeed, it was shown that wrestlers have the motivation to achieve success and avoid failure, and are not motivated by external prizes [32].

Moreover, a study on Portuguese national Olympic team wrestlers investigated motivation (according to the SDT motivation continuum) related to recovery processes in wrestlers, as recovery is related to better results and sports performance [33]. They recorded a positive relationship between intrinsic motivation and recovery process dimensions of social and personal well-being, indicating that wrestlers practice in voluntary, satisfying, and pleasurable ways. Their findings indicate that intrinsically motivated athletes are more prone to commit, learn, and persist in their sports careers [33]. Therefore, more intrinsically motivated wrestlers would be more committed to recovery processes in an enjoyable, fun, and pleasurable way, which would consequently lead to better sports performance.

To further support our findings, the sports motivation profile of athletes involved in sports other than wrestling will be compared. Previously, an Irish study investigated sports motivation using the SMS-II in athletes aged 18–35 years from team sports, including basketball, soccer, hockey, hurling, and rugby [34]. They noted that their athletes had intrinsic motivation scores of 4.93 ± 1.4, which is significantly lower than our wrestlers, who had scores of 5.97 ± 0.90. Additionally, other self-determined forms of motivation were lower in the Irish team athletes than in our wrestlers: integrated regulation (4.81 ± 1.38 vs. 5.99 ± 0.83) and identified regulation (4.76 ± 1.48 vs. 6.08 ± 0.82) [34]. Moreover, a Finnish study on team sport adolescent athletes reported that elite athletes have higher intrinsic motivation and lower amotivation compared to non-elite athletes [35]. In a study of football players aged 13–20 years, more autonomous motivation was linked to positive results in a perceived effort that promoted task-involvement climate and basic psychological needs satisfaction of players [36]. Additionally, young handball players aged 16–17 years displayed high self-determined motivation coupled with a high task-involving climate, high basic psychological needs, and commitment [37]. Track and field athletes aged 13–18 displayed high levels of intrinsic motivation, with males and athletes from urban living environments having higher levels compared to females and rural athletes [38]. From this brief overview of motivation profiles in several sports, it could be supported that self-determined forms of motivation are crucial for sports success. Additionally, according to the comparison between different types of sports (team vs. individual/combat), it can be stated that wrestlers indeed do possess a high level of self-determined motivation, which is proven to be related to better performance and persistence in practicing sports [24].

Somewhat opposite to our findings, a study on Hungarian national team wrestlers reported that younger wrestlers aged 10–19 years mainly relied on external regulation, but this trend was not present in the older age group (19–25 years) [39]. Additionally, amotivation was high in the youngest age group, while it had a descending pattern through older age groups [39]. However, we investigated athletes from the older age group, and we can assume that they already had their self-determined types of motivation formed on the higher levels. Moreover, a study on Croatian wrestlers aged 18.5 ± 3.58 years stated that younger wrestlers (cadets) most likely have lower positive intrinsic motivation (i.e., interest, enjoyment, and perceived competence), and higher negative intrinsic motivation (pressure) compared with older athletes (juniors and seniors) [40]. The authors of that study used different scale for assessing motivation (Intrinsic Motivation Inventory); thus, the results are hard to compare.

### 4.2. Differences in Motivation Profile between Successful and Less Successful Wrestlers

Our results showed that successful wrestlers had significantly higher intrinsic motivation than less successful wrestlers. Supportive to our findings, a study investigating Russian elite and intermediate wrestlers aged 18.4 ± 4.84 years evidenced that elite wrestlers are more intrinsically motivated than less successful wrestlers [41]. Therefore, the observed difference in the most self-determined form of motivation (i.e., intrinsic motivation) adds to the previous part of the discussion. This additionally proves that wrestlers are driven by internal factors, such as personal satisfaction and joy, and are not motivated by external prizes.

Observing from the aspect of the country that wrestlers are from, the predominance of self-determined motivation can indeed be explained by personal enjoyment and success, and not being motivated by the prize (e.g., money). In Croatia, wrestling is not a mainstream sport and is not highly financially supported by the governing bodies. Additionally, the salary of a competitive wrestler is substantially lower than in other sports (e.g., football, basketball, and handball). For example, the best wrestlers who participated in the highest level of competitive wrestling (i.e., Olympic games) have a very low salary and are usually forced to work while they compete, which makes their sports path even harder. However, they have the honor to, for example, hand out medals at national competitions and promote wrestling on mass media (i.e., television). Collectively, according to our results and from our personal knowledge of the situation, wrestlers in Croatia are mainly training and competing to fulfill their satisfaction and joy, and are not motivated by external prizes.

Findings from previous studies that coaches’ behavior influences athletes’ motivation should be considered as an important guideline for coaches. Previously, a study on Turkish elite freestyle and Greco-Roman wrestlers recorded a positive relationship between intrinsic motivation and wrestlers’ perception of instruction and the training behavior of their coaches [42]. Supportively, a study on Croatian athletes noted that coaches can increase athletes’ intrinsic motivation by demonstrating training and instruction behavior, social support, and positive feedback behavior [43]. Additionally, a study on college athletes involved in various sports (i.e., football, tennis, gymnastics, volleyball, basketball, and track and field) investigated the relationship between coaching behaviors and athletes’ intrinsic motivation [44]. Study revealed that all coaching behaviors, including autocratic and democratic behavior, positive feedback, training, and instruction, predicted the perceived competence, autonomy, and intrinsic motivation of their athletes [44]. Therefore, the results of this research emphasize that coaches could additionally try to develop a more self-determined motivation of their wrestlers. This way, they could influence changing attitudes, behavior, persistence in sports, and overall sports performance of youth wrestlers.

### 4.3. Limitations and Strengths

This research has several limitations. The main limitation comes from the cross-sectional type of the study. This means that the causality could not be determined, and the results should be interpreted with caution. For example, it is possible that performance predicted motivation, meaning that successful wrestlers were more motivated, as winning is motivational. However, this research emphasizes the importance of creating a positive and self-determined motivational climate among wrestlers, regardless of the bidirectional possibility of interpreting the results. Thus, the main strength of this study is that it investigated one really important psychological construct that forms the wrestler’s actions and enables optimal sports performance. Another important thing is that we included youth wrestlers, who can still be guided, and the coach greatly influences their development. Therefore, this research can be used as a guideline for coaches to create and nurture motivation among their youth athletes.

## 5. Conclusions

Youth Greco-Roman wrestlers possess high levels of self-determined motivation, which allows them to endure the suffering in combat and the high psycho-physiological demands of both training and competitions. Moreover, successful wrestlers have higher levels of intrinsic motivation than less successful wrestlers, which points out that wrestlers should be intrinsically motivated to achieve good competitive success. Investigating and determining sport motivation is very helpful for practitioners and coaches that strive to optimize their athletes’ performance and well-being. It enables coaches to engage and connect with their athletes’ motivation, which consequently leads to better performance and well-being. Future research should investigate wrestlers from other age groups (both younger and older) to completely determine the sport motivation profile of wrestlers and enable their optimal sports development.

## Figures and Tables

**Table 1:** Internal consistency of SMS-II subscales.

	Cronbach’s Alpha	Inter-Item Correlation
Amotivation	0.58	0.32
External regulation	0.66	0.41
Introjected regulation	0.33	0.18
Identified regulation	0.72	0.47
Integrated regulation	0.75	0.51
Intrinsic motivation	0.79	0.57

**Table 2:** Descriptive statistics and differences according to performance categories.

	Total Sample	Successful (*n* = 27)	Less Successful (*n* = 20)	ES
	Mean ± SD	Mean ± SD	Mean ± SD	
Age (years)	17.71 ± 1.62	17.96 ± 1.34	17.41 ± 1.89	0.34
Training experience (years)	6.89 ± 2.75	7.46 ± 2.69	6.19 ± 2.73	0.47
Competing experience (years)	6.45 ± 2.83	6.96 ± 2.73	5.81 ± 2.89	0.41
Competition ranking	4.07 ± 2.97	2.04 ±0.87	7.18 ± 2.24	3.22
Body weight (kg)	77.73 ± 14.31	79.31 ± 15.93	76.03 ± 12.43	0.23
Body height (cm)	177.45 ± 7.33	177.26 ± 7.30	177.64 ± 7.50	0.05
Body mass index	24.53 ± 3.23	25.03 ± 3.42	23.98 ± 2.97	0.32
Body fat percentage	15.43 ± 6.54	14.90 ± 6.99	16.00 ± 6.11	0.17

Note: SD-standard deviation, ES-Cohen’s d effect size.

**Table 3:** MANOVA for the motivation and anthropometric/body composition variables.

Factor	Wilks’ Lambda	F	*p*
Motivation	0.77	2.01	0.09
Anthropometric/body composition	0.87	1.80	0.15

## Data Availability

Not applicable.

## References

[1] Chaabene H., Negra Y., Bouguezzi R., Mkaouer B., Franchini E., Julio U., Hachana Y. (2017). Physical and Physiological Attributes of Wrestlers: An Update. J. Strength Cond. Res..

[2] Marques V., Coswig V., Viana R., Leal A., Alves F., Alves A., Teles G., Vieira C., Silva M., Santos D. (2019). Physical Fitness and Anthropometric Measures of Young Brazilian Judo and Wrestling Athletes and Its Relations to Cardiorespiratory Fitness. Sports.

[3] Horswill C.A. (1992). Applied physiology of amateur wrestling. Sport. Med..

[4] García-Pallarés J., López-Gullón J.M., Muriel X., Díaz A., Izquierdo M. (2011). Physical fitness factors to predict male Olympic wrestling performance. Eur. J. Appl. Physiol..

[5] Cieśliński I., Gierczuk D., Sadowski J. (2021). Identification of success factors in elite wrestlers-An exploratory study. PLoS ONE.

[6] Issurin V.B. (2017). Evidence-Based Prerequisites and Precursors of Athletic Talent: A Review. Sports Med..

[7] Gould D., Dieffenbach K., Moffett A. (2002). Psychological Characteristics and Their Development in Olympic Champions. J. Appl. Sport Psychol..

[8] Dimundo F., Cole M., Blagrove R.C., Till K., Kelly A.L. (2022). A Multidisciplinary Investigation into the Talent Development Processes in an English Premiership Rugby Union Academy: A Preliminary Study through an Ecological Lens. Sports.

[9] Lochbaum M., Zanatta T., Kirschling D., May E. (2021). The Profile of Moods States and Athletic Performance: A Meta-Analysis of Published Studies. Eur. J. Investig. Health Psychol. Educ..

[10] Morgan W.P. (1980). Test of champions: The iceberg profile. Psychol. Today.

[11] Nagle F.J., Morgan W.P., Hellickson R.O., Serfass R.C., Alexander J.F. (1975). Spotting Success Traits in Olympic Contenders. Physician Sportsmed..

[12] Turksoy A., Güvendi B., Sahin M., Korkmaz M. (2016). Determining the relationship between self-efficacy, perception of success and motivation in junior national wrestling team athletes. Int. Refereed Acad. J. Sport..

[13] Clancy R.B., Herring M.P., MacIntyre T.E., Campbell M.J. (2016). A review of competitive sport motivation research. Psychol. Sport Exerc..

[14] Roberts G.C., Treasure D. (2012). Advances in Motivation in Sport and Exercise.

[15] Pelletier L.G., Rocchi M.A., Vallerand R.J., Deci E.L., Ryan R.M. (2013). Validation of the revised sport motivation scale (SMS-II). Psychol. Sport Exerc..

[16] Deci E.L., Ryan R.M. (1985). Cognitive evaluation theory. Intrinsic Motivation and Self-Determination in Human Behavior.

[17] Vallerand R.J., Losier G.F. (1994). Self-determined motivation and sportsmanship orientations: An assessment of their temporal relationship. J. Sport Exerc. Psychol..

[18] Pelletier L.G., Tuson K.M., Fortier M.S., Vallerand R.J., Briere N.M., Blais M.R. (1995). Toward a new measure of intrinsic motivation, extrinsic motivation, and amotivation in sports: The Sport Motivation Scale (SMS). J. Sport Exerc. Psychol..

[19] Rodrigues F., Pelletier L., Rocchi M., Cid L., Teixeira D., Monteiro D. (2021). Adaptation and Validation of a Portuguese Version of the Sports Motivation Scale-II (SMS-II-P) Showing Invariance for Gender and Sport Type. Percept. Mot. Ski..

[20] Frederick-Recascino C.M. (2002). Self-determination theory and participation motivation research in the sport and exercise domain. Handbook of Self-Determination Research.

[21] Standage M., Ryan R.M. (2020). Self-determination theory in sport and exercise. Handbook of Sport Psychology.

[22] Connelly L.M. (2011). Cronbach’s alpha. Medsurg Nurs..

[23] Adeyeye F., Vipene J., Asak D. (2013). The impact of motivation on athletic achievement: A case study of the 18th National Sports Festival, Lagos, Nigeria. Acad. Res. Int..

[24] Gillet N., Vallerand R.J., Amoura S., Baldes B. (2010). Influence of coaches’ autonomy support on athletes’ motivation and sport performance: A test of the hierarchical model of intrinsic and extrinsic motivation. Psychol. Sport Exerc..

[25] Stenling A., Lindwall M., Hassmén P. (2015). Changes in perceived autonomy support, need satisfaction, motivation, and well-being in young elite athletes. Sport Exerc. Perform. Psychol..

[26] Keshtidar M., Behzadnia B. (2017). Prediction of intention to continue sport in athlete students: A self-determination theory approach. PLoS ONE.

[27] Almagro B.J., Sáenz-López P., Moreno-Murcia J.A., Spray C. (2015). Motivational Factors in Young Spanish Athletes: A Qualitative Focus Drawing From Self-Determination Theory and Achievement Goal Perspectives. Sport Psychol..

[28] Mosqueda S., López-Walle J.M., Gutiérrez-García P., García-Verazaluce J., Tristán J. (2019). Autonomous Motivation as a Mediator Between an Empowering Climate and Enjoyment in Male Volleyball Players. Sports.

[29] Berki T., Piko B.F., Page R.M. (2019). The Relationship Between the Models of Sport Commitment and Self-Determination among Adolescent Athletes. Acta Fac. Educ. Phys. Univ. Comen..

[30] Domuschieva-Rogleva G. (2015). Determinant of sport motivation with wrestling athletes. Res. Kinesiol..

[31] Fuentes C.A., Gullón J.M.L., Belmonte M.J.B., Ferri J.M.V., Sánchez S.A., Berenguí R. (2020). Psychological dimension in the formation process of the spanish olympic wrestler. An. Psicol./Ann. Psychol..

[32] Korobeynikov G., Mazmanian K., Korobeynikova L., Jagiello W. (2011). Diagnostics of psychophysiological states and motivation in elite athletes. Bratisl. Lek. Listy.

[33] Martins P., Pedro S. (2017). Motivational Regulations and Recovery in Olympic Wrestlers. Int. J. Wrestl. Sci..

[34] Sheehan R.B., Herring M.P., Campbell M.J. (2018). Associations Between Motivation and Mental Health in Sport: A Test of the Hierarchical Model of Intrinsic and Extrinsic Motivation. Front. Psychol..

[35] Zanatta T., Rottensteiner C., Konttinen N., Lochbaum M. (2018). Individual Motivations, Motivational Climate, Enjoyment, and Physical Competence Perceptions in Finnish Team Sport Athletes: A Prospective and Retrospective Study. Sports.

[36] Monteiro D., Teixeira D.S., Travassos B., Duarte-Mendes P., Moutão J., Machado S., Cid L. (2018). Perceived Effort in Football Athletes: The Role of Achievement Goal Theory and Self-Determination Theory. Front. Psychol..

[37] Alesi M., Gómez-López M., Chicau Borrego C., Monteiro D., Granero-Gallegos A. (2019). Effects of a Motivational Climate on Psychological Needs Satisfaction, Motivation and Commitment in Teen Handball Players. Int. J. Environ. Res. Public Health.

[38] Chin N.S., Khoo S., Low W.Y. (2012). Self-determination and goal orientation in track and field. J. Hum. Kinet..

[39] Szemes Á., Vig P., Nagy K., Géczi G., Sipos K., Tóth L. (2017). Age-related differences in motivational climate and extrinsic-intrinsic motivational factors among members of the Hungarian national wrestling teams. Cogn. Brain Behav..

[40] Slačanac K., Baić M., Karninčić H. (2021). The relationship between rapid weight loss indicators and selected psychological indicators on success of Croatian wrestlers. Arch. Budo.

[41] Grushko A., Bochaver K., Shishkina A., Kabanov D., Konstantinova M., Vavaev A., Kasatkin V. (2016). Psychological and psychophysiological profile in combat sports. Rev. Artes Marciales Asiáticas.

[42] Sarı İ., Bayazıt B. (2017). The Relationship between Perceived Coaching Behaviours, Motivation and Self-Efficacy in Wrestlers. J. Hum. Kinet..

[43] Barić R., Bucik V. (2009). Motivational differences in athletes trained by coaches of different motivational and leadership profiles. Kinesiology.

[44] Hollembeak J., Amorose A.J. (2005). Perceived Coaching Behaviors and College Athletes’ Intrinsic Motivation: A Test of Self-Determination Theory. J. Appl. Sport Psychol..

